# Assessing pooled BAC and whole genome shotgun strategies for assembly of complex genomes

**DOI:** 10.1186/1471-2164-12-194

**Published:** 2011-04-15

**Authors:** Niina Haiminen, F Alex Feltus, Laxmi Parida

**Affiliations:** 1 IBM T.J. Watson Research Center, P.O. Box 218, Yorktown Heights, NY 10598, USA; 2 Department of Genetics & Biochemistry, Clemson University, Clemson, SC 29634, USA; 3 Clemson University Genomics Institute, Clemson University, Clemson, SC 29634, USA

## Abstract

**Background:**

We investigate if pooling BAC clones and sequencing the pools can provide for more accurate assembly of genome sequences than the "whole genome shotgun" (WGS) approach. Furthermore, we quantify this accuracy increase. We compare the pooled BAC and WGS approaches using *in silico *simulations. Standard measures of assembly quality focus on assembly size and fragmentation, which are desirable for large whole genome assemblies. We propose additional measures enabling easy and visual comparison of assembly quality, such as rearrangements and redundant sequence content, relative to the known target sequence.

**Results:**

The best assembly quality scores were obtained using 454 coverage of 15× linear and 5× paired (3kb insert size) reads (15L-5P) on *Arabidopsis*. This regime gave similarly good results on four additional plant genomes of very different GC and repeat contents. BAC pooling improved assembly scores over WGS assembly, coverage and redundancy scores improving the most.

**Conclusions:**

BAC pooling works better than WGS, however, both require a physical map to order the scaffolds. Pool sizes up to 12Mbp work well, suggesting this pooling density to be effective in medium-scale re-sequencing applications such as targeted sequencing of QTL intervals for candidate gene discovery. Assuming the current Roche/454 Titanium sequencing limitations, a 12 Mbp region could be re-sequenced with a full plate of linear reads and a half plate of paired-end reads, yielding 15L-5P coverage after read pre-processing. Our simulation suggests that massively over-sequencing may not improve accuracy. Our scoring measures can be used generally to evaluate and compare results of simulated genome assemblies.

## Background

Strategies for effectively sequencing entire large genomes typically employ one of two approaches: A) BAC-by-BAC Sanger sequencing of clones that represent a minimum tile path (MTP) derived from a physical map (e.g. HICF, high information content fingerprinting, [1]) as has been carried out for rice and maize [2,3]; or B) a whole-genome shotgun (WGS) sequencing paradigm in which genomic libraries are sequenced using the Sanger technique as was done for, e. g., *Populus*, grapevine, and sorghum [4-6]. The MTP approach requires library preparation and the construction of a physical map which increases assembly accuracy at an additional cost. However, a prime advantage to using an MTP and individual BAC clones relative to a WGS strategy is that assembly errors are localized to individual sequenced clones and the incorrect pasting of distal chimeric regions of the genome is prevented.

With the advent of next-generation sequencing technologies, it has become possible to sequence genomic DNA at high coverage and quickly relative to Sanger-based methods. For example, the Roche/454 pyrosequencing approach has been shown to be effective in sequencing genomic DNA fragments from several species including barley [7-9], rice [10], and Atlantic salmon [11]. Given the reduction in time and cost offered by new sequencing technologies, it is now possible to modify the MTP strategy by pooling BAC clones and "cheaply" sequencing them at sufficient coverage to provide for accurate assembly. Pooling speeds up the sequencing process and dramatically reduces cost. This approach was taken by Rounsley et al. [10]; they used Roche (454) Titanium next-generation sequencing reads of 6 BAC pools of ~3Mbp MTP each to assemble a 19Mbp region of the short arm of rice chromosome 3 [10]. In our study, we sought to extend the findings of Rounsley et al. and others by using a simulation approach. We constructed MTPs from several plant genomes and assembled them using simulated Roche/454 reads with injected sequencing error.

Choosing the best sequencing strategy has received attention recently [12,13]. Schatz *et al. *[13] discussed the tradeoffs among read length, coverage, and expected contig length in a genome assembly. They also reviewed recently published genome assemblies generated with various second-generation sequencing strategies. Gnerre *et al. *[12] briefly explored the effect of using different read coverage levels in a mouse chromosome assembly. Additionally, Goldberg *et al. *[14] have determined proportions of conventional Sanger vs. 454 sequencing data that would yield high-quality yet cost-effective assemblies.

Our goal in this work was to A) implement a scoring system to determine assembly accuracy through re-alignment with a reference genome; B) determine which sequencing coverage yields optimal assembly results on an *A. thaliana *3Mbp pool; C) test whether the pooled BAC approach could be extended beyond 3Mbp pools; and D) determine if the approach would work on genomes of varying complexity.

## Results and discussion

### Construction of simulated reads

Simulated 454 sequencing reads were generated for each genome sequence studied. Read lengths and quality scores were extracted from actual 454 sequencing data generated from *T. cacao *(see Materials and methods). Models for generating uneven sequencing coverage and sequencing errors were developed and applied (see Materials and methods). The error rates applied correspond to published estimates [9], with insertion/deletion type errors being more frequent than nucleotide substitutions, and homopolymer runs being especially prone to errors. Each BAC pool consists of a collection of BACs organized as a MTP. The MTP organization of the BACs was extracted from actual data available in the MSU rice database [15], and is characterized in Materials and methods. BAC end sequences (BES) like those that would be produced as a result of Sanger sequencing were generated for each pool's minimum tiling path, and additional BES were generated once per every 20 kbp, on average, across the reference sequence. Sanger-sequencing type errors were also introduced into the BES.

### Scoring functions

We developed a scoring system to use in evaluating each assembled pseudomolecule against the reference sequence. Each score has a value from 0 (lowest quality) to 1 (perfect match). Higher scores indicate better pseudomolecule sequence quality with respect to matching regions, relocations, inversions, and redundancy. We chose these features as an intuitive and comprehensive set that captures the effects of correct and erroneous assembly. Coverage of the reference and various assembly size statistics are often used to evaluate assemblies. Here we also focus on the misassembled regions; we are able to capture these since we know the reference sequence. Our scoring functions can be applied to any genome comparison and provides a common reference point in evaluating assembly algorithms. Details on the scoring functions are given in Materials and methods.

### Optimizing linear and shotgun read mixtures for various *Arabidopsis *BAC pool sizes

The effect of mixing 5X coverage increments of linear (L) and paired (P) 454 read mixtures on 3Mbp, 6Mbp, 9Mbp, 12Mbp *Arabidopsis *genome pools was tested. All combinations, up to 20X coverage (see Additional file 1), were assembled using 'default' or 'optimized' CABOG assembly parameters (see Materials and methods). Dot-plot alignments between the template and assembly pseudomolecules under both assembly conditions are shown in Additional file 2 while numerical score comparisons of the same pseudomolecules are shown in Table 1. Assembler statistics for the 3 Mbp and 12 Mbp pool assemblies can be found in Additional file 3 and even more complete accounting, including data for the 6 Mbp and 9 Mbp pool sizes, is shown in Additional file 4.

**Table 1:** Comparison of default and optimized CABOG assembly parameters

Default	Match	Relocation	Inversion	Redundancy	Coverage	Scaffold N50	Number of scaffolds
*Arabidopsis thaliana*	0.527	0.987	0.954	0.962	87.44%	117,128	57
*Vitis vinifera*	0.524	0.994	0.955	0.982	84.77%	114,280	60
*Oryza sativa*	0.535	0.992	0.966	0.982	88.49%	112,776	39
*Populus trichocarpa*	0.529	1.000	0.984	0.980	82.74%	134,082	65
*Sorghum bicolor*	0.542	0.889	0.944	0.956	86.58%	148,894	37
*Zea mays*	0.506	0.872	0.734	0.835	76.38%	52,719	140

**Optimized**	**Match**	**Relocation**	**Inversion**	**Redundancy**	**Coverage**	**Scaffold N50**	**Number of scaffolds**

*Arabidopsis thaliana*	0.645	0.996	0.996	0.979	99.86%	891,801	12
*Vitis vinifera*	0.694	0.993	0.953	0.992	99.46%	1,578,318	5
*Oryza sativa*	0.723	0.997	0.911	0.996	99.79%	1,785,140	6
*Populus trichocarpa*	0.813	1.000	1.000	0.997	99.16%	2,338,043	6
*Sorghum bicolor*	0.674	0.973	0.998	0.989	97.15%	663,137	11

Assembly evaluation scores and scaffold statistics determined for pseudomolecules created using 3 Mbp pools with 15x linear + 5x paired read coverage are shown for all of the genomes studied. Scores were determined using default and optimized assembly parameters, except for *Z. mays *that was only assembled using default parameters.

**Figure 1:**
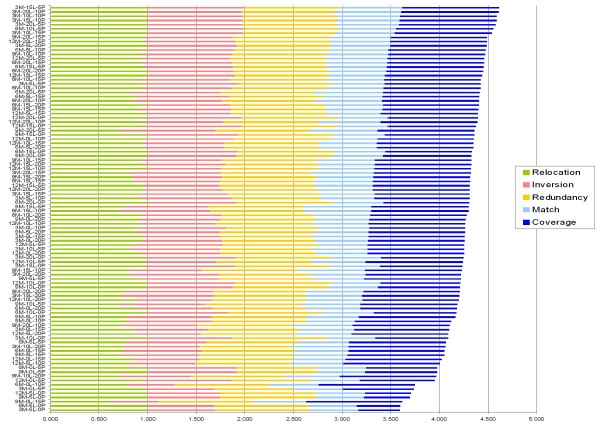
***Arabidopsis *assembly scores**. Alignment scores as compared to the template sequence are shown for all 4 BAC pool sizes (3M = 3Mbp, 6M = 6Mbp, 9M = 9Mbp, 12M = 12Mbp) and for all read mixture combinations (L = linear reads; P = paired reads). Bars are sorted by the sum of all scores.

**Figure 2:**
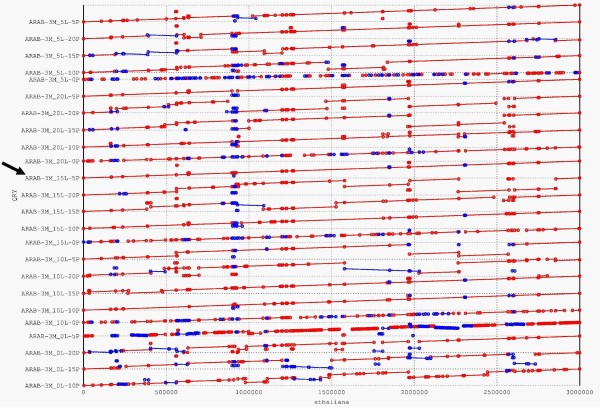
**Dot plots of *Arabidopsis *assemblies**. Alignments of the 3 Mbp Arabidopsis pseudomolecules compared to the reference sequence are shown for all 24 read mixture combinations (L = linear reads; P = paired reads). Red dots and lines denote matching regions in the correct orientation, blue denotes reversed orientation. Longer lines denote larger matching regions between the pseudomolecule and the reference sequence. The 15L-5P assembly with best quality scores is indicated with an arrow.

Measures of assembly correctness were obtained using our scoring functions, all five scores for the *Arabidopsis *assemblies are shown in Figure 1. The assembly with the best combined score was obtained using a 15X linear (15L) and 5X paired (5P) mixture derived from a 3Mbp pool (Figure 1). The best read mixtures for the 6Mbp, 9Mbp, and 12Mbp pools were 10L-5P, 20L-15P, and 20L-15P, respectively. The assembly scores for all of the *Arabidopsis *pool mixtures can be found in Additional file 5. Dot-plots of 3 Mbp assemblies obtained using the 'optimized' assembly parameters graphically revealed that various read mixes can inhibit proper assembly (Figure 2). For example, the 3Mbp assembly with the worst score, showing a strikingly poor assembly in Figure 2, was derived from a 5L-0P mix.

When initiating an expensive genome sequencing project, it is important to decide the minimum amount of sequence information required for an assembly of desired quality. Our simulations indicate that there is an optimal sequencing depth required for obtaining an accurate assembly that covers a high percentage of the target genome sequence. Under-sequencing is intuitively an obvious problem which can be visualized using our methods (e.g. 3Mbp 5L-0P and 0L-5P in Figures 1 &2). Less intuitively, over-sequencing can inhibit assembly quality as well as evidenced by the 3 Mbp 20L-20P assembly being ranked 18^th ^out of the twenty-four 3Mbp assemblies analyzed (Figure 1). We also found that 15L-5P was sufficient to yield a 3Mbp super-scaffold of reasonable quality making it an excellent target sequence mix and depth for a 3Mbp pooled BAC project; however, the addition of 5L + 15P would double the sequencing cost and diminish the assembly quality of such a project, as suggested by the low rank of the 20L-20P assembly in Figure 1. Furthermore, coverage can be reduced even further if a lower-quality draft is sufficient for applications such as whole-genome comparisons with a high-quality reference genome.

### Exploring assembly scores vs. coverage

**Figure 3:**
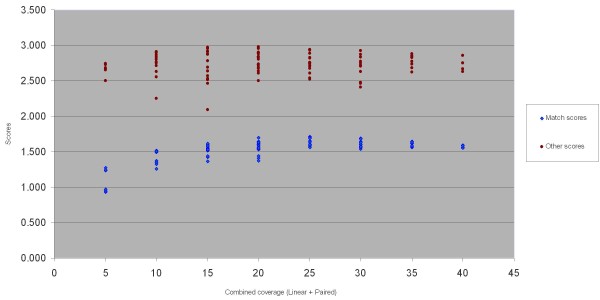
**Assembly scores vs. combined coverage for *Arabidopsis *assemblies**. Assembly scores are divided into two categories: match scores (M, C) and other scores (RL, I, RD). Their combined values are shown for all 3—12 Mbp Arabidopsis assemblies, as a function of linear plus paired coverage.

To better understand the dependencies between read coverage and assembly scores, we studied their relationships in detail. We divided the scores into two categories: match scores (M, C) and other scores (RL, I, RD), and studied the relationship between scores and total read coverage. Figure 3 shows the results for all *Arabidopsis *3-12 Mbp pools. These results indicate that increasing read coverage up to a certain saturation point (25X as observed from Figure 3) improves match scores; after that point increasing coverage may not improve the results. The other scores stay more or less constant across read coverages.

**Figure 4:**
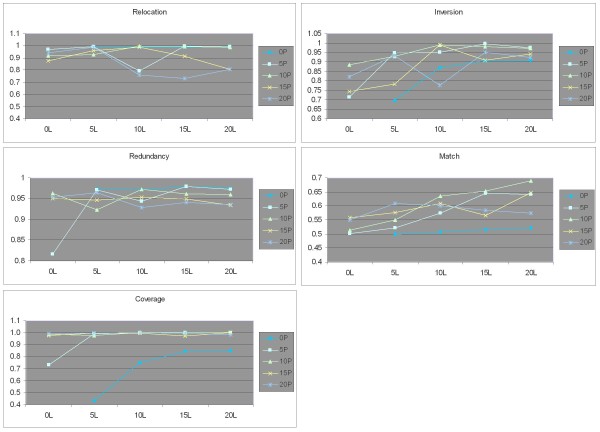
**Assembly scores vs. coverage for *Arabidopsis *3 Mbp assemblies**. The value of each score is detailed as a function of linear coverage. The lines represent constant paired coverage, and show the change in scores when increasing linear coverage.

We also took a closer look at the behavior of all individual scores for the 3 Mbp *Arabidopsis *assemblies, the results are shown in Figure 4. Each line represents a fixed paired coverage, with each x-axis point represents a linear coverage. One can for example see that the best relocation score at 0L linear coverage is achieved with 5P paired coverage, and that the match score at 10P paired coverage always improves when linear coverage increases. This detailed look at the assembly scores shows that adding coverage does not always produce better results, and explains which aspects of some of the high-coverage assemblies contribute to poor scores compared to lower-coverage assemblies.

### Comparing assemblies among six plant genomes

**Figure 5:**
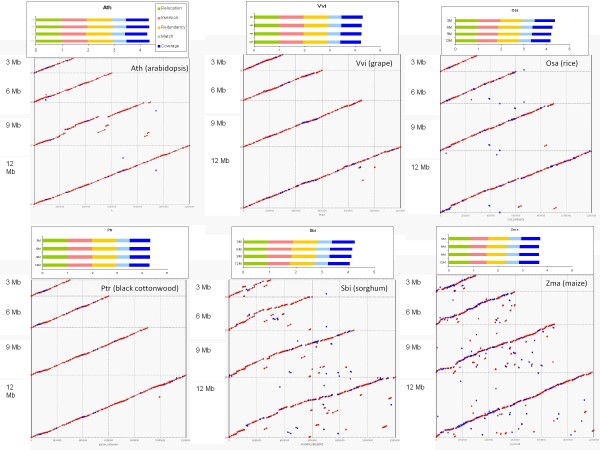
**Dot plots of *non-Arabidopsis *genome assemblies**. Alignments of 3 Mbp, 6 Mbp, 9 Mbp and 12 Mbp pseudomolecules against the reference sequences are shown for coverage 15x linear + 5x paired. Studied genomes are *V. vinifera *(Vvi), *O. sativa *(Osa), *P. trichocarpa *(Ptr), *S. bicolor *(Sbi), and *Z. mays *(Zma). Red dots and lines denote matching regions in the correct orientation, blue denotes reversed orientation. The associated assembly scores are also shown.

In order to verify that our simulation results are applicable to other genomes, we simulated additional 3,6,9,12 Mbp paired and linear datasets derived from the grape ([4]; *Vitis vinifera*), rice ([2]; *Oryza sativa*), black cottonwood ([6]; *Populus trichocarpa*), sorghum ([5]; *Sorghum bicolor*), and maize ([16]; *Zea mays*) genome assemblies. Differences between these genomes are outlined in Table 2, and the studied sequences are detailed in Additional file 6. These five genomes represent two additional dicot and three monocot genomes comprised of very different GC and repeat sequence contents. We then assembled the simulated sequence data using the 15L-5P mix that had been optimal for the *Arabidopsis *3 Mbp assembly (Table 3, Figure 5). The scores we obtained for these assemblies are comparable to those we obtained in the *Arabidopsis *simulation using the 15L-5P mix (Table 3) indicating that while our assembly results vary somewhat from genome to genome, reasonable assemblies of reasonably good quality can be reliably generated using the 15L-5P mix. Additional file 7 contains all CABOG assembly statistics, including N50 and gap statistics, for each assembly in our simulation study.

**Table 2:** Comparison of genomes used in the simulation study

	Version Used for Statistics	Chrs	Scaffolds > 10Kbp	Genome Assembly Size	GC Content	Repeat Content
*Arabidopsis thaliana*	TAIR 9	5	5	119,146,348	35.97%	16.51%
*Vitis vinifera*	Genoscope 12X	19	19	486,198,630	33.41%	47.25%
*Populus trichocarpa*	JGI v2	19	794	417,137,944	32.57%	18.98%
*Oryza sativa*	MSU v6.1	12	12	372,317,567	43.55%	38.70%
*Sorghum bicolor*	JGI sb1	10	19	738,540,932	41.49%	63.12%
*Zea mays*	MGP 4a.53	10	10	2,061,021,377	46.60%	73.06%

Repeat fraction was determined by counting the fraction of masked nucleotides from http://www.phytozome.net (V6.0) masked assemblies except for Ath1 which was taken from the Premasked Genome (araTha5) from http://www.repeatmasker.org/PreMaskedGenomes.html.

**Table 3:** Pooled BAC assembly scores for five genomes

Species	Pool Size	Match	Relocation	Inversion	Redundancy	Coverage	Scaffold N50	Number of scaffolds
*A. thaliana*	3M	0.527	0.994	0.954	0.962	87.44%	117,128	57
*A. thaliana*	6M	0.518	0.997	0.976	0.969	86.55%	126,619	100
*A. thaliana*	9M	0.511	0.927	0.985	0.975	85.72%	121,006	144
*A. thaliana*	12M	0.510	0.999	0.981	0.982	87.36%	147,618	152
*V. vinifera*	3M	0.524	0.997	0.955	0.988	84.76%	114,280	60
*V. vinifera*	6M	0.511	0.997	0.974	0.972	80.60%	113,117	143
*V. vinifera*	9M	0.510	0.997	0.953	0.969	82.96%	116,543	173
*V. vinifera*	12M	0.507	0.994	0.941	0.973	82.48%	115,403	226
*O. sativa*	3M	0.535	0.996	0.966	0.989	88.45%	112,776	39
*O. sativa*	6M	0.519	0.983	0.895	0.983	87.10%	140,891	90
*O. sativa*	9M	0.508	0.961	0.902	0.977	84.91%	117,728	141
*O. sativa*	12M	0.507	0.958	0.869	0.979	84.72%	114,523	219
*P. trichocarpa*	3M	0.529	1.000	0.984	0.981	82.68%	134,082	65
*P. trichocarpa*	6M	0.513	1.000	0.980	0.992	82.31%	119,756	114
*P. trichocarpa*	9M	0.508	1.000	0.995	0.992	81.39%	124,798	182
*P. trichocarpa*	12M	0.506	1.000	0.979	0.989	80.99%	117,268	256
*S. bicolor*	3M	0.542	0.945	0.944	0.957	86.56%	148,894	37
*S. bicolor*	6M	0.520	0.889	0.939	0.955	86.03%	147,854	81
*S. bicolor*	9M	0.508	0.922	0.898	0.953	84.59%	112,136	213
*S. bicolor*	12M	0.504	0.905	0.859	0.953	83.57%	96,480	274
*Z. mays*	3M	0.506	0.872	0.734	0.835	76.38%	52,719	140
*Z. mays*	6M	0.503	0.857	0.679	0.832	79.31%	40,526	320
*Z. mays*	9M	0.502	0.847	0.687	0.820	80.33%	37,394	485
*Z. mays*	12M	0.501	0.855	0.674	0.815	82.39%	37,122	577

Evaluation scores and scaffold statistics for assemblies determined using 15x linear + 5x paired read coverage and pool sizes 3 Mbp, 6 Mbp, 9 Mbp, and 12 Mbp are shown for all of the genomes studied. Default assembly parameters were used.

### Assembly simulations of whole genomes: whole genome shotgun vs. 3 Mbp pooled BAC approaches

We studied the differences in whole genome assembly results obtained using the WGS and pooled BAC approaches. Read coverages of 15x linear + 5x paired were used. All five *Arabidopsis *chromosomes were first modeled as an MTP of BACs (the same MTP structure as was used for the previous 3 Mbp pools). For the WGS approach, reads were generated across the chromosomes and provided to the assembler. The resulting assembly consisted of pseudomolecules for each of the five chromosomes. For the pooled approach, the MTP was divided into pools of approximately 3 Mbp and each pool's reads were assembled independently. Thirty-nine pseudomolecules, one for each pool, were subsequently assembled. The pools for each chromosome were combined to produce chromosome-scale pseudomolecules.

**Figure 6:**
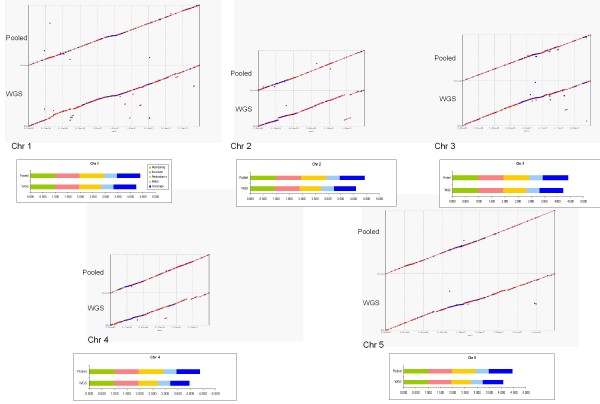
**Dot plots of *Arabidopsis *chromosome assemblies**. Alignments of pseudomolecules are shown against the sequences of each of the *Arabidopsis *chromosomes; analyses using whole genome shotgun (WGS) sequencing (bottom half of each dot plot) and 3 Mbp pooled (Pooled) sequencing (top half of each dot plot) are both shown. Coverage combination of 15x linear + 5x paired was used for both analyses. Red dots and lines denote matching regions in the correct orientation, blue denotes reversed orientation. The associated assembly scores are also shown.

**Figure 7:**
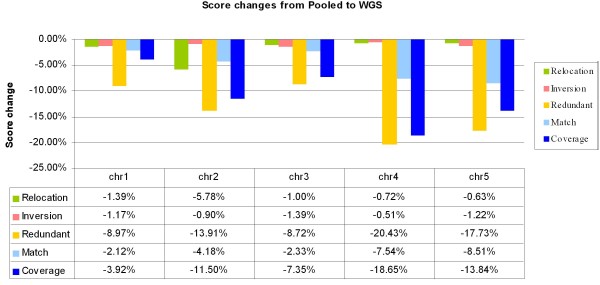
**Pooled vs. WGS *Arabidopsis *chromosome scores**. Relative changes in assembly scores when going from pooled to WGS assembly are shown for each *Arabidopsis *chromosome.

Dot plots of the chromosome-scale pseudomolecules are shown in Figure 6. These pseudomolecules were scored against the reference chromosome sequences. The scores are provided in Table 4, and scores for individual pools are provided in Additional file 8. In Figure 6, a few major relocation errors were observed for the WGS approach (e.g., large off-diagonal segment in chromosome 2). Closer examination of the relative changes in individual WGS scores with respect to the pooled BAC scores (Figure 7) revealed that the pooled BAC approach improves all assembly scores, as expected. A great improvement was observed in coverage, e.g., increase from 76% with the WGS method to 93% with the pooled BAC approach for chromosome 4. We achieved only 76—90% coverage per chromosome using the WGS approach but 93—97% coverage per chromosome, a definite improvement, using the pooled BAC approach. The redundancy score was also clearly better for pooled BACS compared to WGS.

**Table 4:** Scores for *Arabidopsis *assemblies determined using the pooled BAC and WGS strategies

	Size (Mbp)	Method	Match	Relocation	Inversion	Redundancy	Coverage	Scaffold N50	Number of scaffolds
chr1	30.4	WGS	0.501	0.986	0.957	0.891	90.42%		
chr2	19.7	WGS	0.496	0.940	0.954	0.846	84.97%		
chr3	23.5	WGS	0.503	0.988	0.939	0.893	90.09%	207,281	3,268
chr4	18.6	WGS	0.479	0.992	0.950	0.778	76.01%		
chr5	30.0	WGS	0.492	0.993	0.955	0.804	83.02%		

chr1	30.4	Pooled	0.512	1.000	0.968	0.979	94.11%	369,269	497
chr2	19.7	Pooled	0.518	0.998	0.962	0.982	96.01%	534,038	291
chr3	23.5	Pooled	0.515	0.998	0.953	0.978	97.23%	464,102	431
chr4	18.6	Pooled	0.518	0.999	0.955	0.977	93.43%	355,538	370
chr5	30.0	Pooled	0.537	1.000	0.967	0.978	96.36%	750,202	390

Assembly evaluation scores and scaffold statistics are shown for the *Arabidopsis *chromosome pseudomolecules obtained from assembling WGS and pooled BAC data. Chromosome scaffold statistics for pooled assemblies are averages over the values for each pool in that chromosome. Optimized assembly parameters were used.

We also examined whether repeated sequence content could have contributed to the lower scores we determined for some assemblies as opposed to others. We compared the *Arabidopsis *repeat sequence content annotated by RepeatMasker [17] to the corresponding assembly scores for each BAC pool. The results are shown in Additional file 8 and Additional file 9. Gradual decrease of assembly scores with increased repeat sequence content was observed, which supports the common understanding that repeat-rich regions of genomes are more challenging to assemble.

### Comparing a single insert size library with multiple insert size libraries with the same combined coverage

Our assemblies thus far are based on various combinations of linear and paired reads, where paired reads arise from a 3 Kbp insert size library. However, multiple insert libraries are critical for assembling whole-genome shotgun libraries in practice. Longer reads tend to bridge repetitive DNA which is difficult to resolve by short reads. However, the fact that sequencing pooled 3Kbp fragments by 454 (paired/linear) alone is sufficient for a reasonable assembly is a useful observation since only a single library construction is required thereby significantly reducing costs.

The fact that more 3Kbp fragments are nested within repeats relative to 8Kbp fragments and we still get good assemblies is an indication of the robustness of the genome measurements made by the mixed linear/paired 3Kbp 454 approach. With that said, we still performed some assemblies with 8Kbp fragments to see if the assembly correctness changes when assembling 5X paired 3Kbp pairs coverage, compared to assembling the same amount of paired coverage but from two different insert size libraries (3Kbp and 8Kbp). Additional 15X linear coverage was used in both cases. The results of this comparison are shown for 3—12 Mbp segments from the maize genome in Table 5. When using two different insert size libraries, coverage improves but relocation and redundancy scores are reduced, indicating more assembly mistakes with the 3Kpb + 8Kbp paired read mixture. The scaffold N50 size also increases when using the two insert size libraries, indicating the assemblies with the 3Kpb + 8Kbp paired read mixture are less fragmented.

**Table 5:** *Zea mays *assembly scroes for a single insert size library versus multiple insert size libraries

Coverage	Pool Size	Match	Relocation	Inversion	Redundancy	Coverage	Scaffold N50	Number of scaffolds
*15L + 5P (3kb)*	3M	0.506	0.872	0.734	0.835	76.38%	52,719	140
*15L + 5P (3kb)*	6M	0.503	0.857	0.679	0.832	79.31%	40,526	320
*15L + 5P (3kb)*	9M	0.502	0.847	0.687	0.820	80.33%	37,394	485
*15L + 5P (3kb)*	12M	0.501	0.855	0.674	0.815	82.39%	37,122	577

*15L + 2.5P (3kb) + 2.5P (8 kb)*	3M	0.511	0.717	0.728	0.818	85.83%	104,440	94
*15L + 2.5P (3kb) + 2.5P (8kb)*	6M	0.504	0.700	0.733	0.799	84.25%	104,094	216
*15L + 2.5P (3 kb) + 2.5P (8 kb)*	9M	0.503	0.752	0.703	0.807	85.54%	76,589	337
*15L + 2.5P (3kb) + 2.5P (8kb)*	12M	0.502	0.815	0.717	0.778	86.36%	69,622	432

Assembly evaluation scores and scaffold statistics are shown for maize 3--12 Mbp pool assemblies constructed from (i) 15X linear + 5X paired 3 Kbp insert size library reads, and (ii) 15X linear + 2.5X paired 3 Kbp insert size library + 2.5X paired 8 Kbp insert size library reads.

## Conclusions

High-throughput sequencing technologies enable the sequencing of genomic DNA at high coverage and much more rapidly than Sanger-based methods. With the decrease in both time and expense it is possible, as well as practical, to pool BAC clones and sequence the pools at coverages that provide for accurate assembly. The obvious advantage of such an approach, over whole genome shotgun sequencing (WGS), is that errors in assembly related to distal chimeric regions are completely avoided and any other errors are restricted to only local regions of the assembly. We have examined this approach for assembling genome sequences of varying complexity and for pool sizes up to 12 Mbp. We have also performed a whole-genome assembly simulation of the 119 Mbp *Arabidopsis *genome and compared the results to results obtained using the WGS approach. To quantify the assembly quality in relation to the different variables of our experiment, we devised measures that enable comparisons of multiple assemblies. We found that although a physical map, or some alternative additional information, is required to order the scaffolds in both the WGS and pooled BAC approaches, the latter results in assemblies of higher quality as determined using our objective quality scores.

 We achieved only 76—90% coverage per chromosome using the WGS approach but 93—97% coverage per chromosome, a definite improvement, using the pooled BAC approach. Since BAC pools of up to 12Mbp were assembled well, and assuming an approximate Roche/454 Titanium limit of 400bp reads and 200Mbp/plate, our results suggest that a 12 Mbp genomic region could be re-sequenced with only a full plate of linear reads and a half plate of paired-end reads, which would yield what we found to be the optimal 5x paired and 15x linear coverage combination after read pre-processing. Our results also indicate that over-sequencing may not yield more accurate results, sequencing beyond 30X coverage is not required and a 15L-5P mix of a 3Mbp DNA fragment library may be adequate to produce a draft genome sequence of good quality.

 We believe that the simulated pooled BAC assemblies turned out to be of sufficient quality for de novo assembly of targeted regions. These assemblies have imperfections like all assemblies, but we feel they are of sufficient quality for gene discovery as well as providing a mapping template for re-sequencing applications. Therefore, we see the pooled-BAC approach targets researchers who require a quality assembly from only a portion of the genome for A) re-sequencing or B) validation of a WGS assembly. Of course, there are limitations to using a partial genome as a template for mapping short reads since homologous reads from distal parts of the genome could map to the sub-genome assembly. However, this problem can still occur with a full genome assembly.

Finally, the scoring measures presented here can be used generally to evaluate and compare results of simulated genome assemblies.

Although factors such as cost and speed have influenced the change of sequencing technologies over the years, challenging the assembly process the algorithms have coped well and more and more genomes are getting sequenced every day. However the nagging worries continue to be accuracy as well as completeness of these genome assemblies. Sequencing technologies are changing rapidly but our basic expectation from them, which is the correctness the genome assembly, continues to remain the same. In this study, for the sake of tractability, we removed the assembler out of the equation to the extent possible and focused on the effect of regimes on the correctness of the sequence assembled. We observed in our experiments that increasing the sequencing depth progressively or introducing multiple libraries did not necessarily improve the quality of the assembled sequence. This lack of monotonicity in the accuracy is counter-intuitive and we can only speculate as to what the possible reasons could be. Thus in this complex multifactorial task of genome sequencing, a natural and reliable approach to examining the effectiveness of different regimes suggested by the underlying technologies and prevalent wisdom, we believe is a controlled (simulation) environment. The community will greatly benefit by the establishment of benchmark set of genomes (or sub-genomes) with a range of varying characteristics such as repeat content and genome sizes, to check the effectiveness of both the technologies as well as the algorithms. In this paper, we used a set of six plant genomes with repeat contents varying from 16% to 73% and size from 119 Mbp to 2 Gbp. We also presented a set of objective (i.e., assembler-independent) correctness scores to evaluate the results. We believe the combination of the benchmark genomes and the assembly evaluation criteria is a good starting point and an effective test-bed even for the technologies of the future.

## Methods

### Construction of simulated datasets

Details of the chosen reference sequences for all of the genomes studied are shown in Additional file 6. Any missing bases (N) in the reference were replaced with A/T or C/G based on the frequencies of these nucleotides in that genome's sequence. Lengths and quality scores for the simulated reads were extracted from actual *T. cacao *454 sequencing data (B Scheffler, unpubl. data). The mean length of unpaired reads was approximately 350 bp and standard deviation 150 bp. The mean length of the paired reads we generated was 170 bp and standard deviation70 bp and was based on estimates of 454 paired read lengths from *T. cacao *(K Mockaitis, pers. comm.).

Simulated reads were generated as follows. To model non-uniformity in the read coverage, each BAC sequence was divided into windows of 100 bp. Each window was first assigned a generating density of max{N(5,1),0}, (where N stands for the normal distribution), after which they were scaled so that all windows' densities sum to 1. Each read was first assigned to a window, probability of the assignment being equal to the window density, and then the read's starting position within the window was chosen randomly.

Substitution errors were introduced at a probability of 0.1% per nucleotide, insertions and deletions each at 0.5% probability per nucleotide, with runs of the same nucleotide (homopolymer runs) of length at least 3 being more prone to errors. These error rates correspond to published estimates on 454 error profiles [9]. In more detail, the error injection protocol is as follows. The read sequence is read one homopolymer run after another (they can be of length 1 or more). If the homopolymer run has length at least 3, it is assigned a higher probability of containing errors than a run of length 1 or 2. We define *totalErrorRate *as the sum of desired substitutions, insertions, and deletion error percentages. The per-nucleotide probability that there is an error in a homopolymer run of length one or two is a*totalErrorRate, while for runs of length at least three the probability is b*totalErrorRate (factors *a *= 0.8 and *b *= 1.6 were empirically chosen to approximately yield *totalErrorRate *= 1.1% in the read collection). If it is determined that a nucleotide has an error, the error type is determined according to the fraction of substitution, insertion, and deletion type errors. The only restriction on error type is, that if an insertion occurs in the homopolymer run, only insertions and substitutions, no deletions, can occur in the remaining positions in the run (and the same for deletions).

Paired reads were generated by first randomly choosing locations for inserts, according to the window density described above. Mean insert size was 3 kbp and std 20 bp. Short sequences from each end of the insert were extracted to generate two reads. The two reads were assigned labels indicating the index of the insert they originated from. Finally, errors were injected to the reads according to the procedure described above.

The minimum tiling path (MTP) BAC structure for the 3 Mbp pool was extracted from an actual rice tiling path (MSU v6.1; [15]). The statistics are as follows: min. BAC size 47 kbp, max. BAC size 191 kbp, mean BAC size 145 kbp, min. overlap between BACs 0.6 kbp, max. overlap between BACs 113 kbp, mean overlap between BACs 36 kbp. The same MTP was concatenated multiple times to cover the larger pools. Additional BACs were generated randomly across the reference sequence and simulated 600—700 bp Sanger sequencing reads were extracted from their ends. The frequency of the additional BAC ends was such that one would be expected to occur every 20 kbp. Substitutions were injected at a rate of 0.006% per nucleotide and insertions and deletions were each introduced with a probability of 0.0002% per nucleotide, in agreement with the estimate of Sanger per base accuracy being as high as 99.999% [18]. Actually any error rate < 0.1% is expected to yield less than one erroneous base for each 600—700 bp BES we simulate.

### Assembly conditions

All assemblies were constructed using the Celera CABOG assembler v6.0 (beta) software [19] on either the Clemson University "Palmetto cluster" distributed computing system with 8856 cores (June 2010; Rmax = 66.18 TFlops: Rpeak = 81.48 20 TFlops) or a Clemson University Genomics Institute 32-core 2.6Ghz AMD machine with 256GB of RAM. Reads were prepared by converting FASTA sequence and quality files into the CABOG FRG format files using the convert-fasta-to-v2.pl (-454 switch) software [19,20]. Assemblies were performed at various read depths and paired-shotgun mixtures using the runCA script under 'default' or 'optimal' conditions in which the following parameters were provided to the assembler: (overlapper = mer; obtOverlapper = mer; ovlOverlapper = mer; unitigger = bog; utgGenomeSize = X = pool size; doToggle = 1).

### Pseudomolecule construction

Scaffold output FASTA files were BLASTN (E < = 1e-75; Percent nucleotide identity > = 98%; [21,22]) aligned to the appropriate MTP BAC end sequence (BES) set. Scaffolds were ordered based upon the first BES order from the MTP and oriented based upon the position of the first and last BES hit along the scaffold. Gaps of 70 X's were inserted between scaffolds.

### Scoring functions

Five characteristics that reflect fundamental aspects of assembly quality were scored: relocation, RL; inversion, I; redundancy, RD; match, M; and coverage, C (defined below). For each score, value 1 is best and 0 is worst. We computed the values of these scores by comparing the assembled pseudomolecule against the known reference sequence using Blast version 2.2.15 (default parameters; [21,22]). BLAST hits that were at least 1 Kbp long were subjected to the following.

Relevant information, as described below, was extracted from the Blast matches and included in a data table T of size n, n being the reference sequence length. If we assume a reference R of length n, and an assembly A of length m, then for each position i, where i = 1,...,n, the value of T[i] is the coordinate of the *closest *position j in the assembly, T[i] = argmin_j_{| i- j |}, such that there is a match between R[i] and A[j]. A match on the reverse strand of the assembly indicates an inversion and the value of T[i] becomes -j. If there does not exist any match for position i, then T[i] = 0. Length l is defined as the number of reference positions that have a match to the assembly being evaluated.

**Relocation score, RL**, accounts for pairs of points that are in an incorrect order in the assembly with regard to the reference sequence. Because performing pairwise comparisons on millions of locations is computationally infeasible, we identify large relocation errors using a sampling approach. Relocation score, RL, is computed as follows. Table T is sampled at x locations (in the experiments reported herein x = 10,000), that is, a point from T is sampled every n/x positions. When the order of two sampled points, a and b, and their values do not agree, e.g. T[a] > T[b] but a < b, the number of disagreements, #d, is increased for both a and b. As there are p = (x ^2 ^- x) possible disagreements, the relocation score is normalized to [0,1] by defining RL = 1 - #d/p.

**Inversion score, I**, denotes the fraction of the matching assembly positions that match the same strand on the reference sequence, inverted positions decrease the score. Inversion score is defined as I = 1 - ∑1_i_/l, where 1_i _= 1 when T[i] < 0 and is 0 otherwise.

**Redundancy score, RD**, penalizes for any unnecessary content in an assembly such as assembled portions of sequence that map to locations that are already covered by other portions of the assembly, and assembled portions of sequence that do not match the reference at all. An additional data table, U, was used for redundancy score computations. Instead of recording the closest assembly position for each reference position, we store the *closest reference position*, U[j] = argmin_i_{| i - j |}, such that there is a match between R[i] and A[j], for each assembly position j. The reference locations that occur exactly once among the entries in U, #u, yields the fraction of unique and useful content of the assembly through the following equation, RD = #u/m.

**Match score, M**, is designed to reward for long contiguous matches and to penalize for gaps. We first segmented the reference length, n, into mutually non-intersecting segments: alternating matching regions, u, (where T[i] != 0) and gap regions, v, (where T[i] = 0): Σ_s_u_s _+ ∑_t_v_t _= n. We assigned a reward, weighted by factor α, for each matching segment, and assigned a penalty, weighted by factor β, for each gap segment. We defined the match score as M = 1/( α + β ) ( α∑_s_(|u_s_|/n)^η1 ^+ β∑_t_(|v_t_|/n)^η2 ^), and we used parameter values η1 = η1 = 2 in our experiments. In determining Match score, M, more emphasis can be placed on either the matches or the gaps by changing the parameter values in this equation.

**Coverage, C**, is the fraction of matches to the reference sequence. C = ∑1_i_/n, where 1_i _= 1 when T[i] ! = 0 and is 0 otherwise.

## Abbreviations

BAC: bacterial artificial chromosome; BES, BAC: end sequence; HICF: high information content fingerprinting; Mbp: megabase pairs; MTP: minimum tile path; WGS: whole genome shotgun.

## Authors' contributions

NH contributed to the experimental design, generated the simulated data, and contributed to developing and implementing the scoring functions. FAF contributed to experimental design, performed the assemblies, and constructed the pseudomolecules. LP contributed to the experimental design and to developing the scoring functions. All authors contributed to preparing the manuscript, and read and approved the final manuscript.

## Supplementary Material

Additional file 1 **Read coverage combinations**. Table of possible read coverage combinations. Click here for file

Additional file 2 **Default vs. optimized assembly parameters**. Figure comparing default and optimized assembly parameters for 3Mbp assemblies of five genomes. Click here for file

Additional file 3 ***Arabidopsis *pooled BAC assembly statistics**. Table with *Arabidopsis *pooled BAC assembly statistics. Click here for file

Additional file 4 **Detailed *Arabidopsis *pooled BAC assembly statistics**. Table showing detailed *Arabidopsis *assembly statistics for 3Mbp and 12Mbp pool assemblies. Click here for file

Additional file 5 ***Arabidopsis *pools' assembly scores**. Table showing assembly evaluation scores for *Arabidopsis *pool assemblies. Click here for file

Additional file 6 **Reference sequences**. Table showing reference sequences and coordinates for studied source genomes. Click here for file

Additional file 7 **CABOG assembly statistics**. Tar archive containing CABOG assembly statistics for each assembly in our simulation study. Click here for file

Additional file 8 ***Arabidopsis *genome pools' assembly scores**. Table showing assembly scores for individual pseudomolecules of each 3Mbp pool for the *Arabidopsis *genome. Click here for file

Additional file 9 ***Arabidopsis *genome pools' repeat content vs. assembly scores**. Figure showing repeat content and scores for *Arabidopsis *whole genome pool assemblies. Click here for file
