# Supplementary material for: Assessing pooled BAC and whole genome shotgun strategies for assembly of complex genomes
Source: BMC Genomics. 2011 Apr 15;12:194. doi: 10.1186/1471-2164-12-194 (PMC3224119; doi:10.1186/1471-2164-12-194)
Supplement: Additional file 1 — Read coverage combinations. Table of possible read coverage combinations. [file 1471-2164-12-194-S1.DOC]

| **Table of possible read coverage combinations.** | | | | | |
| --- | --- | --- | --- | --- | --- |
|  |  |  |  |  |  |
| **3M-pool** | **20X (pair)** | **15X (pair)** | **10X (pair)** | **5X (pair)** | **0X (pair)** |
| **20X (linear)** | **20L-20P** | **20L-15P** | **20L-10P** | **20L-5P** | **20L-0P** |
| **15X (linear)** | **15L-20P** | **15L-15P** | **15L-10P** | **15L-5P** | **15L-0P** |
| **10X (linear)** | **10L-20P** | **10L-15P** | **10L-10P** | **10L-5P** | **10L-0P** |
| **5X (linear)** | **5L-20P** | **5L-15P** | **5L-10P** | **5L-5P** | **5L-0P** |
| **0X (linear)** | **0L-20P** | **0L-15P** | **0L-10P** | **0L-5P** |  |
|  |  |  |  |  |  |
| **6M-pool** | **20X (pair)** | **15X (pair)** | **10X (pair)** | **5X (pair)** | **0X (pair)** |
| **20X (linear)** | **20L-20P** | **20L-15P** | **20L-10P** | **20L-5P** | **20L-0P** |
| **15X (linear)** | **15L-20P** | **15L-15P** | **15L-10P** | **15L-5P** | **15L-0P** |
| **10X (linear)** | **10L-20P** | **10L-15P** | **10L-10P** | **10L-5P** | **10L-0P** |
| **5X (linear)** | **5L-20P** | **5L-15P** | **5L-10P** | **5L-5P** | **5L-0P** |
| **0X (linear)** | **0L-20P** | **0L-15P** | **0L-10P** | **0L-5P** |  |
|  |  |  |  |  |  |
| **9M-pool** | **20X (pair)** | **15X (pair)** | **10X (pair)** | **5X (pair)** | **0X (pair)** |
| **20X (linear)** | **20L-20P** | **20L-15P** | **20L-10P** | **20L-5P** | **20L-0P** |
| **15X (linear)** | **15L-20P** | **15L-15P** | **15L-10P** | **15L-5P** | **15L-0P** |
| **10X (linear)** | **10L-20P** | **10L-15P** | **10L-10P** | **10L-5P** | **10L-0P** |
| **5X (linear)** | **5L-20P** | **5L-15P** | **5L-10P** | **5L-5P** | **5L-0P** |
| **0X (linear)** | **0L-20P** | **0L-15P** | **0L-10P** | **0L-5P** |  |
|  |  |  |  |  |  |
| **12M-pool** | **20X (pair)** | **15X (pair)** | **10X (pair)** | **5X (pair)** | **0X (pair)** |
| **20X (linear)** | **20L-20P** | **20L-15P** | **20L-10P** | **20L-5P** | **20L-0P** |
| **15X (linear)** | **15L-20P** | **15L-15P** | **15L-10P** | **15L-5P** | **15L-0P** |
| **10X (linear)** | **10L-20P** | **10L-15P** | **10L-10P** | **10L-5P** | **10L-0P** |
| **5X (linear)** | **5L-20P** | **5L-15P** | **5L-10P** | **5L-5P** | **5L-0P** |
| **0X (linear)** | **0L-20P** | **0L-15P** | **0L-10P** | **0L-5P** |  |
