# Supplementary figures and images for: Assessing pooled BAC and whole genome shotgun strategies for assembly of complex genomes
Source: BMC Genomics. 2011 Apr 15;12:194. doi: 10.1186/1471-2164-12-194 (PMC3224119; doi:10.1186/1471-2164-12-194)

## Slide 1
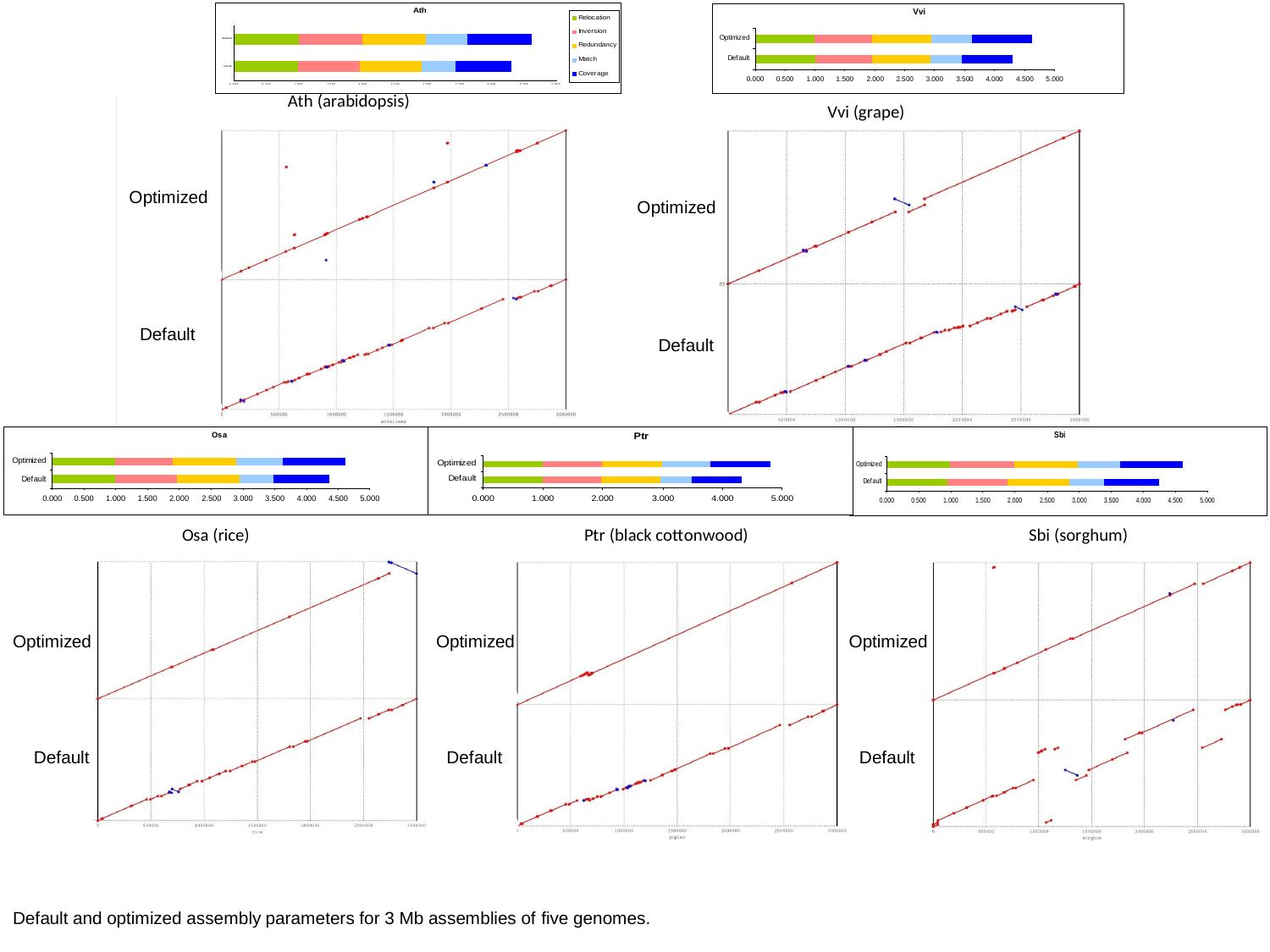

Supplement: Additional file 2 — Default vs. optimized assembly parameters. Figure comparing default and optimized assembly parameters for 3Mbp assemblies of five genomes. [file 1471-2164-12-194-S2.PPT]

## Slide 1
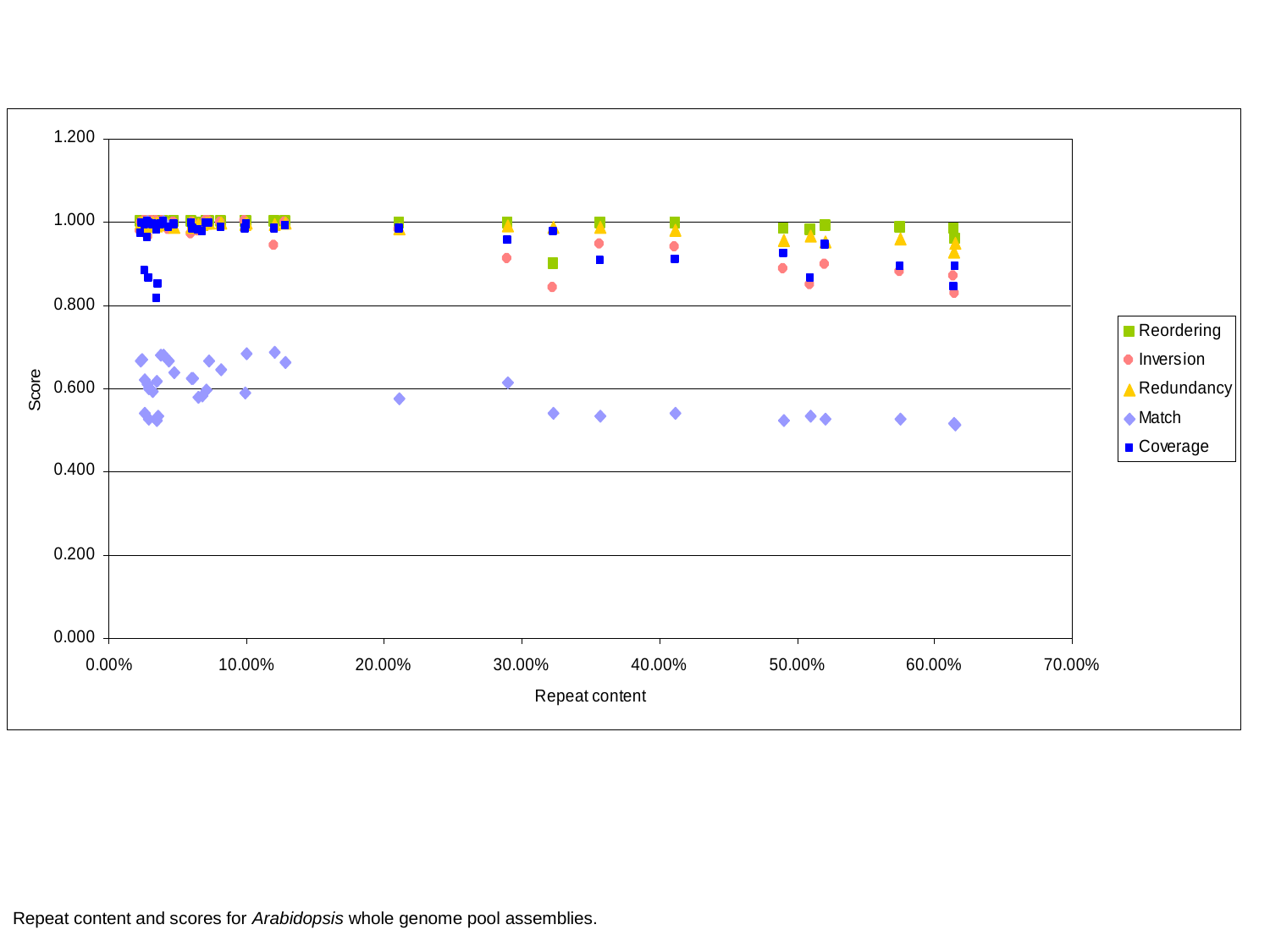

#

Supplement: Additional file 9 — Arabidopsis genome pools' repeat content vs. assembly scores. Figure showing repeat content and scores for Arabidopsis whole genome pool assemblies. [file 1471-2164-12-194-S9.PPT]
