# Supplementary material for: Assessing pooled BAC and whole genome shotgun strategies for assembly of complex genomes
Source: BMC Genomics. 2011 Apr 15;12:194. doi: 10.1186/1471-2164-12-194 (PMC3224119; doi:10.1186/1471-2164-12-194)
Supplement: Additional file 6 — Reference sequences. Table showing reference sequences and coordinates for studied source genomes. [file 1471-2164-12-194-S6.DOC]

| **Studied genome sequences and coordinates** | | | |
| --- | --- | --- | --- |
| **Genome** | **Region** | **Start coordinate** | **NCBI Reference / Source** |
| *Arabidopsis thaliana* | Chr 2 | 4,000,001 | NC_003071.7 |
| *Vitis*  *vinifera* | Vvi18_WGA173_1 | 1 | NW_002238137.1 |
| *Oryza*  *sativa* | Chr 4 | 18,000,001 | NC_008397.1 |
| *Populus trichocarpa* | LG 2 | 1,000,001 | NC_008468.1 |
| *Sorghum bicolor* | Chr 1 | 10,000,001 | <ftp://ftp.jgi-psf.org/pub/JGI_data/phytozome/>  v5.0/Sbicolor/assembly/Sbi1/ |
| *Zea*  *mays* | Chr 10 | 3,000,001 | ftp://ftp.ensemblgenomes.org/pub/plants/release-7/fasta/zea_mays/dna/ |
