# Supplementary material for: Assessing pooled BAC and whole genome shotgun strategies for assembly of complex genomes
Source: BMC Genomics. 2011 Apr 15;12:194. doi: 10.1186/1471-2164-12-194 (PMC3224119; doi:10.1186/1471-2164-12-194)
Supplement: Additional file 8 — Arabidopsis genome pools' assembly scores. Table showing assembly scores for individual pseudomolecules of each 3Mbp pool for the Arabidopsis genome. [file 1471-2164-12-194-S8.DOC]

| **Assembly scores for individual pseudomolecules of each 3 Mbp pool for the Arabidopsis genome.** | | | | | | | | |
| --- | --- | --- | --- | --- | --- | --- | --- | --- |
| **Assembly** | **Method** | **Reordering** | **Inversion** | **Redundancy** | **Match** | **Coverage** | **Repeats** | **Satellite Repeats** |
| chr1_pool_1 | 3Mbp Pool +MTP | 1.000 | 0.985 | 0.990 | 0.611 | 96.18% | 2.84% | 0.00% |
| chr1_pool_2 | 3Mbp Pool +MTP | 1.000 | 0.997 | 0.995 | 0.533 | 85.04% | 3.59% | 0.00% |
| chr1_pool_3 | 3Mbp Pool +MTP | 1.000 | 0.970 | 0.988 | 0.624 | 99.90% | 6.01% | 0.01% |
| chr1_pool_4 | 3Mbp Pool +MTP | 0.999 | 0.994 | 0.996 | 0.685 | 99.31% | 10.06% | 0.11% |
| chr1_pool_5 | 3Mbp Pool +MTP | 0.982 | 0.848 | 0.965 | 0.533 | 86.50% | 51.02% | 10.21% |
| chr1_pool_6 | 3Mbp Pool +MTP | 0.997 | 0.939 | 0.982 | 0.541 | 91.15% | 41.21% | 3.43% |
| chr1_pool_7 | 3Mbp Pool +MTP | 1.000 | 0.998 | 0.998 | 0.644 | 98.84% | 8.22% | 0.00% |
| chr1_pool_8 | 3Mbp Pool +MTP | 0.997 | 0.978 | 0.993 | 0.579 | 98.05% | 6.49% | 0.00% |
| chr1_pool_9 | 3Mbp Pool +MTP | 0.997 | 1.000 | 0.998 | 0.680 | 99.64% | 3.94% | 0.00% |
| chr1_pool_10 | 3Mbp Pool +MTP | 0.999 | 0.972 | 0.986 | 0.540 | 88.33% | 2.66% | 0.12% |
| chr2_pool_1 | 3Mbp Pool +MTP | 0.998 | 0.945 | 0.988 | 0.535 | 90.73% | 35.77% | 0.07% |
| chr2_pool_2 | 3Mbp Pool +MTP | 0.986 | 0.879 | 0.959 | 0.526 | 89.20% | 57.58% | 1.79% |
| chr2_pool_3 | 3Mbp Pool +MTP | 1.000 | 0.998 | 0.997 | 0.663 | 98.93% | 12.86% | 0.11% |
| chr2_pool_4 | 3Mbp Pool +MTP | 1.000 | 1.000 | 0.999 | 0.598 | 99.77% | 7.14% | 0.03% |
| chr2_pool_5 | 3Mbp Pool +MTP | 1.000 | 1.000 | 0.997 | 0.594 | 99.32% | 3.19% | 0.00% |
| chr2_pool_6 | 3Mbp Pool +MTP | 1.000 | 0.978 | 0.999 | 0.666 | 97.42% | 2.37% | 0.03% |
| chr3_pool_1 | 3Mbp Pool +MTP | 1.000 | 0.999 | 0.997 | 0.670 | 99.86% | 2.41% | 0.01% |
| chr3_pool_2 | 3Mbp Pool +MTP | 1.000 | 1.000 | 0.997 | 0.618 | 97.98% | 3.53% | 0.00% |
| chr3_pool_3 | 3Mbp Pool +MTP | 1.000 | 0.995 | 0.997 | 0.681 | 99.41% | 3.84% | 0.00% |
| chr3_pool_4 | 3Mbp Pool +MTP | 0.999 | 0.979 | 0.982 | 0.574 | 98.42% | 21.09% | 0.10% |
| chr3_pool_5 | 3Mbp Pool +MTP | 0.959 | 0.828 | 0.950 | 0.514 | 89.44% | 61.57% | 0.71% |
| chr3_pool_6 | 3Mbp Pool +MTP | 0.898 | 0.839 | 0.987 | 0.540 | 97.66% | 32.29% | 0.19% |
| chr3_pool_7 | 3Mbp Pool +MTP | 1.000 | 0.998 | 0.986 | 0.639 | 99.31% | 4.78% | 0.00% |
| chr3_pool_8 | 3Mbp Pool +MTP | 0.999 | 0.982 | 0.994 | 0.668 | 98.81% | 4.40% | 0.02% |
| chr4_pool_1 | 3Mbp Pool +MTP | 0.998 | 0.912 | 0.990 | 0.615 | 95.64% | 28.98% | 3.94% |
| chr4_pool_2 | 3Mbp Pool +MTP | 0.985 | 0.868 | 0.927 | 0.516 | 84.55% | 61.41% | 4.42% |
| chr4_pool_3 | 3Mbp Pool +MTP | 1.000 | 1.000 | 0.996 | 0.590 | 98.34% | 9.92% | 0.01% |
| chr4_pool_4 | 3Mbp Pool +MTP | 0.998 | 0.987 | 0.995 | 0.582 | 97.69% | 6.82% | 0.00% |
| chr4_pool_5 | 3Mbp Pool +MTP | 1.000 | 0.962 | 0.990 | 0.527 | 86.66% | 2.90% | 0.00% |
| chr4_pool_6 | 3Mbp Pool +MTP | 1.000 | 0.999 | 0.999 | 0.621 | 99.45% | 2.61% | 0.09% |
| chr5_pool_1 | 3Mbp Pool +MTP | 1.000 | 1.000 | 0.999 | 0.601 | 99.89% | 2.91% | 0.03% |
| chr5_pool_2 | 3Mbp Pool +MTP | 1.000 | 0.994 | 0.990 | 0.524 | 81.59% | 3.51% | 0.00% |
| chr5_pool_3 | 3Mbp Pool +MTP | 0.998 | 0.997 | 0.996 | 0.625 | 98.44% | 6.11% | 0.03% |
| chr5_pool_4 | 3Mbp Pool +MTP | 0.984 | 0.885 | 0.957 | 0.524 | 92.29% | 49.04% | 7.15% |
| chr5_pool_5 | 3Mbp Pool +MTP | 0.991 | 0.897 | 0.951 | 0.528 | 94.66% | 52.12% | 4.44% |
| chr5_pool_6 | 3Mbp Pool +MTP | 1.000 | 0.943 | 0.994 | 0.688 | 98.50% | 12.08% | 0.12% |
| chr5_pool_7 | 3Mbp Pool +MTP | 1.000 | 1.000 | 0.998 | 0.667 | 99.82% | 7.26% | 0.04% |
| chr5_pool_8 | 3Mbp Pool +MTP | 1.000 | 1.000 | 0.998 | 1.000 | 100.00% | 4.01% | 0.05% |
| chr5_pool_9 | 3Mbp Pool +MTP | 1.000 | 1.000 | 1.000 | 1.000 | 100.00% | 0.028 | 0.24% |
